# Suicide Literacy and Stigmatising Attitude Among Poisoning Wards Nurses and Physicians Towards Patients With Suicide Attempts: A Cross‐Sectional Study

**DOI:** 10.1002/nop2.70182

**Published:** 2025-04-30

**Authors:** Hanieh Omrani Tabari, Zohreh Hosseini Marznaki, Sussan Moudi, Mobin Alijanzadeh Kashi, Afsane Fendereski, Nipin Kalal, Andrew Fournier, Murat Yıldırım, Aliasghar Manouchehri

**Affiliations:** ^1^ Babol University of Medical Sciences Babol Iran; ^2^ Imam Ali Hospital at Amol City Mazandaran University of Medical Sciences Sari Iran; ^3^ Health Research Institute, Babol University of Medical Sciences Babol Iran; ^4^ Department of Biostatistics, Faculty of Health Mazandaran University of Medical Sciences Sari Iran; ^5^ College of Nursing, all India Institute of Medical Sciences Jodhpur Rajasthan India; ^6^ National Park Service Tucson Arizona USA; ^7^ Department of Psychology, Faculty of Science and Letters Agri Ibrahim Cecen University Ağrı Türkiye; ^8^ Psychology Research Center Khazar University Baku Azerbaijan; ^9^ Clinical Research Development Center, SHahid Beheshti Hospital Babol University of Medical Sciences Babol Iran

**Keywords:** nurses, physicians, suicide knowledge, suicide stigma

## Abstract

**Aim:**

Health professionals' attitudes towards suicidal patients may affect the quality of treatment and care provided. This study was conducted to investigate the knowledge and attitudes of physicians and nurses towards patients with suicidal ideation and a history of suicide attempts.

**Design:**

A cross‐sectional research design was used to conduct this study.

**Methods:**

A total of 421 physicians and nurses were recruited. Data analyses were performed using SPSS software version 23. This study used a sociodemographic questionnaire, the Stigma of Suicide Scale‐Short Form and the Literacy of Suicide Scale questionnaires.

**Results:**

This study included 421 participants, comprising 55 general physicians (GPs; 13.1%), 92 specialist physicians (21.9%) and 274 nurses (65%). Nurses had an average score of 4.65 ± 1.78 for suicide knowledge and 46.59 ± 7.43 for suicide stigma. No significant relationship was observed between suicide knowledge and suicide stigma among nurses (*r* = 0.02). However, a significant negative correlation was identified between suicide knowledge and suicide stigma among physicians (*r* = −0.25).

**Conclusion:**

These findings suggest that increasing suicide literacy may help reduce stigma among physicians, highlighting the need for targeted educational interventions in clinical settings.

**Patient or Public Contribution:**

Patients and the public were not directly involved in the design, conduct or reporting of this study. However, the findings highlight the importance of improving healthcare professionals' knowledge and attitudes towards suicidal ideation and behaviour, which could have a direct impact on patient care.

## 1 Introduction

Suicide is a critical public health issue, particularly among those aged 15 to 45, where it is the second leading cause of death globally. Healthcare professionals' (HCPs) understanding of suicide, or “suicide literacy,” is key to providing effective care and support. Early identification of at‐risk individuals requires awareness of the causes, risk factors and signs of suicide (Lew et al. 2022). Socio‐cultural, economic and psychological factors influence suicide (Manouchehri et al. 2022). Economic disparity, familial strife, mental health disorders and abuse contributed to the 60% rise in suicides in Iran from 2015 to 2019 (Asadiyun and Daliri 2023; Manouchehri et al. 2022; Rasanah 2020). Many suicide attempts, often involving self‐poisoning, lead to hospitalisations, a trend seen both in Iran and globally (Ziapour et al. 2023).

Physicians and nurses provide both mental and physical care to suicide patients. They also understand that awareness of suicidal ideation impacts treatment outcomes (Jandial et al. 2021). Nurses, who spend more time with patients than other HCPs, play a key role in shaping the quality of care (Rizzo et al. 2023). However, nurses who hold negative views towards patients who have attempted suicide contribute to inferior treatment (Jones et al. 2015; Kishi et al. 2011; Ouzouni and Nakakis 2009). Such attitudes are linked to insufficient training in suicide prevention, fostering stigma and discrimination, and lowering care standards Suicidal behaviour progresses through a spectrum. The low end of the spectrum starts from thoughts of suicide (expressed verbally or non‐verbally) to planning, attempting and completion (Wasserman et al. 2012). Suicide literacy requires awareness of warning signs, underlying causes, risk factors, available treatments and prevention strategies (Batterham et al. 2013).

Mental health problems often carry stigma (Al‐Shannaq and Aldalaykeh 2021; Lathabhavan et al. 2024; Masoomi et al. 2023; Özaslan et al. 2024; Özaslan and Yıldırım 2021). Such stigmatising beliefs are widely held about people with suicidal thoughts, especially in low‐ and middle‐income countries (Al‐Shannaq and Aldalaykeh 2023; Kishi et al. 2011; Maruf et al. 2022; Rukundo et al. 2022). Negative societal perceptions of suicide may cause adverse reactions in the victims and their relatives (Carpiniello and Pinna 2017). Reactions include heightened distress, lowered self‐confidence and experiencing discrimination. Such reactions reduce willingness to seek help (Calear et al. 2014; Carpiniello and Pinna 2017; Scocco et al. 2016). A lack of understanding or simple recognition of mental health conditions may contribute to the development of stigmatising attitudes. Conversely, greater awareness among HCPs can lessen stigma and encourage a greater readiness to seek treatment for psychological distress (Jung et al. 2017; Reavley and Jorm 2014).

Emphasis on the importance of seeking mental health assistance serves to prevent further mental health decline and increased risk of suicide. Researchers did find a negative relationship between personal stigma and future intention of seeking help. However, the same researchers did not find that public stigma predicted a lower intention of seeking help (Lally et al., 2013). Research indicated that only 62% of individuals who attempted suicide sought mental health treatment in the year before their attempt (Noorani et al. 2017). Yet, psychiatrists' emotional responses to patient suicide and suicide attempts vary (Alshutwi et al. 2023).

Mental health stigma poses a barrier to the use of mental health care in Iran (Taghva et al. 2017). Individuals encountering apathetic HCPs may not take advantage of health services (Tavakoli et al. 2010). Improving nurses' knowledge and suicide literacy will ultimately make seeking help seem more attractive (Gholamrezaei et al. 2019). Studies revealed that when it comes to suicide, HCPs' views and knowledge differ. Some researchers revealed that HCPs had a more positive outlook and higher knowledge levels. Yet other studies have found that HCPs have negative attitudes and lower levels of suicide literacy (Jandial et al. 2021). Empathetic HCPs encourage the public to seek help for mental health issues. As the first point of contact for suicide prevention and treatment, they act as “gatekeepers” (Jandial et al. 2021). HCP attitudes towards suicidal behaviour are critical for intervention, and negative attitudes can hinder treatment (Ouzouni and Nakakis 2009).

Research indicates that the quality of nursing care can be affected by multiple factors. Such factors were nurses' knowledge about suicide, risk assessment skills, professional experience and attitudes or beliefs regarding suicide (Jones et al. 2015). Awareness‐raising initiatives are effective in enhancing suicide literacy among medical students and nursing professionals. Some initiatives include targeted workshops, short lectures and comprehensive educational programmes (Nebhinani et al. 2013). Improving mental health literacy towards individuals with suicidal tendencies is linked to improvements in seeking professional psychological help. More importantly, Jandial et al. (2021) suggested that further studies are needed in other cultural contexts, particularly if there is a larger social condemnation of suicide. Therefore, a study is needed to evaluate the knowledge and attitudes of physicians and nurses towards patients with suicidal ideation and a history of suicide attempts.

This study is grounded in Question, Persuade and Refer (QPR) model and Signal Detection Theory. Originally developed for radar use during World War II, QPR is now applied in psychology to understand decision‐making under uncertainty. In identifying suicide warning signs, HCPs may either respond or not, depending on their perception of the signs. Identified biases in this process. Biases include the belief that people who express suicidal intentions rarely act on them, conservative bias (failing to respond to weak signs) and liberal bias (overreacting to all signs). However, in the present study, the researchers propose that other biases may exist due to the cultural context.

Understanding how HCPs perceive and interact with patients is crucial, as these perceptions impact recovery, treatment adherence and future suicide risk. This study aims to assess HCPs' attitudes and knowledge to improve patient care and reduce the stigma surrounding suicide attempts. Previous research has been limited by methodological flaws (Jandial et al. 2021; Masoomi et al. 2023). In Iran, a high stigma around mental health services deters individuals from seeking support. The study will focus on physicians' and nurses' attitudes towards patients with suicidal ideation and past suicide attempts.

## 2 Method

### 2.1 Study Design

This study was a cross‐sectional, descriptive study.

### 2.2 Settings and Sampling

Babol City is a northern city in Mazandaran province in Iran. There are four hospitals affiliated with Babol University of Medical Sciences. After receiving ethical approval, the researcher went to the directorates of the respective hospitals. After receiving written consent from the directors of the hospitals and nursing managers, the researcher went to the wards of all four hospitals and met with the managers of each [emergency, intensive care unit (ICU), internal and post‐ICU] ward. Because the current study employed a formula sampling method, the researchers determined the optimal sample size required to ensure 80% statistical power for detecting a medium effect size with an alpha level of 0.05. Through power analysis using G*Power software, the ideal sample size was calculated to be around 384 participants. To account for potential non‐responses and attrition, we set an initial target sample size of 402 participants (Nebhinani et al. 2013).

The sample size was determined using the following formula at a 95% confidence level.

$$n=\frac{Z_{1-\frac{\alpha }{2}}\times \sigma^{2}}{d^{2}}$$

Based on previous studies and an accuracy factor of 1.5, the initial calculation suggested a sample size of 402 participants. After accounting for an estimated 5% dropout rate, 421 samples were collected (Jandial et al. 2021).

The research team collected data in person from all nurses and physicians who were present in the selected wards (Emergency, ICU, Internal Medicine and Post‐ICU). To ensure thorough coverage of the target population, data were gathered from three wards in each of the four participating hospitals. By using this method, a thorough representation of medical personnel tending to patients who had made suicide attempts during the research period was ensured. There was no sampling, for all of the nurses and physicians who worked in the wards (Emergency, ICU, Internal, Post ICU) of the four hospitals affiliated with Babol University of Medical Sciences were included in the study. Patients who have attempted suicide are treated and cared for by the nurses and physicians in these units.

### 2.3 Data Collection

In this study, the research team collected the data directly from the respondents. To start data collection, the qualified nurses and physicians went to a quiet room. Then, a research team member gave the patients the necessary explanations and the research purpose. Afterwards, a separate research team member read each question to all nurse and physician participants. The research team member annotated responses in the desired column. The process took between 20 and 25 min to complete each questionnaire.

The questionnaire was distributed among the nurses and physicians following the endorsement from the Ethics and Research Committee from August 2022 to September 2023. Each nurse received all the information about the study and questionnaire. Individuals interested in participating were directed to endorse the consent form, complete the questionnaire and submit the documents to the researchers. Data collection spanned three months, during which meticulous efforts were made to ensure thorough and inclusive participation. The response rate of the participants was 97%.

### 2.4 Inclusion and Exclusion Criteria

The criteria for inclusion in the study were that participants had to be from within the cadre of nurses and physicians working in the emergency, ICU, internal and post‐ICU wards. Including nurses and physicians across morning, evening and night shifts, the study targeted individuals holding a minimum of a Bachelor's degree for nurses and a minimum of a GP for physicians, exhibiting a willingness to engage in the research, possessing a service tenure of at least six months working in the emergency, ICU, internal and post‐ICU wards, and an absence of mental illness. Nurses and physicians unavailable for data collection during the specified period were deliberately excluded from participation. Additionally, nurses and physicians who did not meet the inclusion criteria were excluded from the study.

### 2.5 Measures

#### 2.5.1 Research Instruments

The research data were collected using a four‐section instrument, including personal and social information questionnaires—the demographic questionnaire, the Literacy of Suicide Scale (LOSS) and the Stigma of Suicide Scale short form (SOSS‐SF).

#### 2.5.2 The Demographic Questionnaire

The demographic questionnaire included age, sex, level of education, type of employment, suicide ward, suicide training, experience in the suicide ward, number of shifts in the month and work experience.

#### 2.5.3 The LOSS

The LOSS was used in this study to assess participants' knowledge about suicide. This questionnaire, originally developed by (Askari et al. 2021), contains 12 items with two response options, “correct” and “incorrect.” It assesses four areas of suicide knowledge, including (a) signs and symptoms of suicide (3 options), (b) causes or nature of suicidal thoughts and behaviours (4 options), (c) risk factors (2 options) and (d) treatment and prevention of suicide (2 options). Correct answers are awarded 1 point, while incorrect answers receive 0 points. The overall score is determined by summing the correct responses. Instead of using classical testing theory, item response theory was employed to evaluate the psychometrics of this scale (Askari et al. 2021). As a result, internal consistency and factor analysis were not conducted. The interpretation of items mainly depended on the difficulty, represented by the mean score of each item, which indicates the percentage of correct answers given by participants. The Arabic version of the LOSS was used in this particular study (Aldalaykeh et al. 2020). To validate the items, 10 faculty members reviewed the questionnaire, and the Content Validity Index (CVI) and Content Validity Ratio (CVR) were calculated for each question. All questions were found to have acceptable validity in terms of necessity, with a high CVI indicating questions that were clear, relevant and straightforward. The validity and reliability were performed based on the internal correlation coefficient using Cronbach's alpha, which was 0.86.

#### 2.5.4 The SOSS‐SF

For assessing participants' stigmatised attitudes towards suicide, the Suicide Attitude Questionnaire (SOSS), designed by Batterham and Collier in 2013 (Batterham et al. 2013), was employed. This questionnaire consists of 16 items divided into three sections measuring suicidal attitudes. The first section examines stigma (8 items), the second section examines isolation or depression (4 items) and the third section assesses normality or admiration of suicide (4 items). Responses are recorded on a 5‐point Likert scale, ranging from 1 (strongly disagree) to 5 (strongly agree).

The SOSS‐SF includes three subscales: stigmatising attitude towards suicide (8 items), attribution of isolation or depression to suicide (4 items) and normalising or glorifying suicide (4 items). Higher scores indicated stronger agreement with the corresponding concepts. The total scores from each subscale were determined by adding all the responses. This study used the Arabic version of the SOSS‐SF (Aldalaykeh et al. 2020). To validate the items, 11 faculty members reviewed the questionnaire, and the CVI and CVR were calculated for each question. All questions were found to have acceptable validity in terms of necessity, with a high CVI indicating questions that were clear, relevant and straightforward. The instrument's reliability was assessed using Cronbach's alpha coefficient to measure internal consistency. The overall instrument showed an internal correlation of 0.89. To check the questionnaire's stability, the same group of subjects completed the questionnaire again under the same conditions two weeks later, and the scores from both administrations were analysed using the Intraclass Correlation Coefficient (ICC), yielding a result of 0.92 for the entire instrument. In this study, the stigma subscale, isolation subscale and glorification subscale had Cronbach's alpha values of 0.85, 0.72 and 0.75, respectively.

### 2.6 Ethical Consideration

Approval was obtained from the Ethics Committee of the REDACTED (IR.MUBABOL.REC.1401.198). Before participation, written informed consent was obtained from all enrolled individuals. Each participant received comprehensive information regarding the study's objectives and procedures, along with assurances concerning preserving their anonymity and the confidentiality of their data. Furthermore, participants were explicitly informed of their voluntary participation and their right to withdraw from the study at any stage without facing any repercussions.

### 2.7 Data Analysis

Data analysis was conducted using Statistical Package for the Social Sciences Software (SPSS, version 23.0). Descriptive statistics were used to summarise the sample's demographic characteristics, including the calculation of frequencies, mean scores and the range of the concepts under investigation in this study. The total scores from each questionnaire were used for further analysis. The Kolmogorov–Smirnov test indicated that the data did not follow a normal distribution. Consequently, non‐parametric tests, namely the Mann–Whitney and Kruskal–Wallis tests, were employed to examine the relationships between variables. Additionally, generalised linear models were used to explore the relationships between the studied variables and the outcomes of suicide knowledge and suicide stigma. All analyses were conducted at a significance level of 0.05.

## 3 Results

### 3.1 Demographic Characteristics of Participants

Of the 420 participants, 55 (13.1%) were GPs, 92 (21.9%) were specialist physicians, and the remaining were nurses. The proportion of male participants among GPs was significantly higher than that among nurses and specialist physicians (*p* = 0.001). Specialist physicians reported higher levels of satisfaction with their income and a longer history of training in dealing with suicidal cases (*p* < 0.001). Nurses had a greater history of serving in departments that handled suicidal patients (*p* < 0.001). The mean age of nurses was 35.52 ± 5.91. The mean age of the GPs was 33.85 ± 9.55. Specialist physicians had a mean age of 47.09 ± 7.88 years. The mean age of specialist physicians was significantly higher than that of the other participants (*p* < 0.001). Nurses had a mean number of monthly shifts of 4.82 ± 28, significantly higher than GPs and specialist physicians, who had mean monthly shifts of 4.55 ± 8.32 and 5.93 ± 6.8, respectively (*p* < 0.001). Additionally, the mean work experience was 12.4 ± 5.8 years for nurses, 8.6 ± 8.6 years for GPs and 15.8 ± 8.7 years for specialist physicians. These differences were statistically significant (*p* < 0.001). The distribution of variables by occupation is presented in Table 1.

**TABLE 1:** Description of individual, social and dependent variables of doctors and nurses.

Variables	Occupation			*p*
	*n* = 274 (nurses)	*n* = 55 (G.P)	*n* = 92 (S.P)	
Sex				0.001
Male	69 (25.2%)	27 (49.1%)	31 (33.7%)	
Female	205 (74.8%)	28 (50.9%)	61 (66.3%)	
Level of education				—
Bachelor's degree	256 (93.4%)	—	—	
Masters	18 (6.6%)	—	—	
Satisfaction of income				< 0.001
No	252 (92%)	47 (85.5%)	57 (62%)	
Yes	22 (8%)	8 (14.5%)	35 (38%)	
Type of employment				< 0.001
Formal	234 (85.4%)	3 (5.5%)	45 (48.9%)	
Informal	31 (11.3%)	18 (32.7%)	25 (27.2%)	
New graduate	9 (3.3%)	16 (29.1%)	12 (13%)	
Others	0 (0%)	18 (32.7%)	10 (10.9%)	
Suicide ward				
Emergency	87 (31.8%)	—	—	
ICU	44 (16.1%)	—	—	
Internal	119 (43.4%)	—	—	
poisoning	21 (7.7%)	—	—	
others	3 (1.1%)	—	—	
Suicide training				< 0.001
No	201 (73.4%)	38 (69.1%)	46 (50%)	
Yes	73 (26.6%)	17 (30.9%)	46 (50%)	
Experience in the suicide ward				< 0.001
No	130 (47.4%)	41 (74.5%)	64 (69.6%)	
Yes	144 (52.6%)	14 (25.5%)	28 (30.4%)	
Age	35.52 ± 5.911	33.85 ± 9.55	47.09 ± 7.88	< 0.001
Number of shifts in a month	28 ± 4.82	8.32 ± 4.55	6.8 ± 5.93	< 0.001
Work experience	12.4 ± 5.80	8.6 ± 8.6	15.8 ± 8.7	< 0.001

*Note:* Qualitative variables were reported as [number (percent Age)] and quantitative variables as [standard deviation ± mean].

### 3.2 Knowledge About Suicide by Occupation

Suicide knowledge was assessed using the 12‐item LOSS questionnaire, with scores ranging from 0 to 12. Among nurses, the mean knowledge score related to suicide was 4.65 ± 1.78, and the median score was 4, with an interquartile range (IQR) of 4–6. The lowest score observed was 0, while the highest score was 12. For GPs, the mean suicide knowledge score was 4.51 ± 1.51, and the median score was 4, with an IQR of 4, 5. The lowest and highest scores recorded for GPs were 1 and 9, respectively. Specialist physicians had a mean suicide knowledge score of 3.80 ± 1.58, and the median score was 4, with an IQR of 3–5. The lowest and highest scores among specialist physicians were 0 and 8, respectively. The Kruskal–Wallis non‐parametric test revealed a statistically significant difference in the mean suicide knowledge scores between physicians and nurses (*p* = 0.001).

### 3.3 Stigma of Suicide by Occupation

Suicide knowledge was assessed using the 12‐item LOSS questionnaire, with scores ranging from 0 to 12. Among nurses, the mean knowledge score related to suicide was 4.65 ± 1.78, and the median score was 4, with an IQR of 4–6. The lowest score observed was 0, while the highest score was 12. For GPs, the mean suicide knowledge score was 4.51 ± 1.51, and the median score was 4, with an IQR of 4–5. The lowest and highest scores recorded for GPs were 1 and 9, respectively. Specialist physicians had a mean suicide knowledge score of 3.80 ± 1.58, and the median score was 4, with an IQR of 3–4. The lowest and highest scores among specialist physicians were 0 and 8, respectively. The Kruskal–Wallis non‐parametric test revealed a statistically significant difference in the mean suicide knowledge scores between physicians and nurses (*p* = 0.001).

### 3.4 Factors Associated With Suicide Knowledge in Nurses

The study examined the association between various study variables and nurses' knowledge of suicide using generalised linear models. Initially, each variable was individually incorporated into a basic model to assess its relationship with suicide knowledge. Subsequently, these variables were collectively included in a multiple model to analyse their associations with suicide knowledge while accounting for other factors. According to the single‐variable model (simple model), older nurses tended to have a higher level of knowledge about suicide. Conversely, nurses who expressed dissatisfaction with their income displayed significantly lower average scores in suicide knowledge compared to their counterparts.

In the multiple model, it was established that when adjusting for other factors, nurses who were not content with their income still exhibited a significantly lower average score in suicide knowledge compared to their peers. However, no statistically significant relationship was found between the age of nurses and suicide knowledge (as depicted in Table 2).

**TABLE 2:** Relationship of individual and social variables with knowledge of suicide in nurse.

Variable	Univariate model		Multiple model	
	B (95% Wald CI)	*p*	B (95% Wald CI)	*p*
Sex				
Male/Female	−0.07 (−0.172, 0.032)	0.181	−0.26 (−0.747, 0.227)	0.295
Age	0.01 (0.003, 0.017)	0.008	0.012 (−0.132, 0.156)	0.866
Level of education				
Bachelor's degree/Masters	0.071 (−0.107, 0.248)	0.434	0.397 (−0.525, 1.319)	0.398
Satisfaction of income				
No/Yes	−0.305 (−0.467, −0.144)	< 0.001	−1.203 (−2.12, −0.285)	0.010
Type of employment				
Formal/New graduated	0.085 (−0.162, 0.332)	0.500	0.186 (−1.176, 1.549)	0.789
Informal/New graduated	0.056 (−0.22, 0.331)	0.693	0.25 (−1.151, 1.651)	0.727
Ward				
Emergency/Poisoning	0.088 (−0.092, 0.269)	0.336	0.259 (−0.612, 1.129)	0.560
ICU/Poisoning	0.113 (−0.082, 0.309)	0.256	0.277 (−0.65, 1.205)	0.558
Internal/Poisoning	0.144 (−0.031, 0.32)	0.107	0.314 (−0.542, 1.171)	0.472
Suicide training				
No/Yes	0.007 (−0.094, 0.107)	0.898	0.124 (−0.397, 0.645)	0.641
Experience in the suicide ward				
No/Yes	0.013 (−0.076, 0.102)	0.773	0.238 (−0.213, 0.69)	0.301
Work experience	0.01 (0.002, 0.017)	0.012	0.023 (−0.125, 0.171)	0.761
Number of shifts in a month	−0.019 (−0.027, −0.01)	< 0.001	−0.066 (−0.12, −0.013)	0.015

Abbreviations: B, regression coefficient; CI, confidence interval.

### 3.5 Factors Associated With Suicide Stigma in Nurses

The study utilised generalised linear models to explore the connections between study variables and the stigma surrounding suicide. Initially, a straightforward approach was used to fit the model, analysing each variable in isolation. Subsequently, all the variables were collectively incorporated into a multiple model to investigate their relationships with suicidal thoughts while considering the influence of other variables. According to the univariate model, it was evident that suicide stigma was significantly more pronounced in men compared to women. Moreover, nurses with formal and contractual employment status exhibited higher levels of suicide stigma compared to their counterparts in the project period. Older nurses and those with more extensive experience also tended to have higher scores in suicide stigma. In the multiple model, when controlling for other factors, suicide stigma was notably higher in nurses with official and contractual employment status compared to those employed during the project period. Surprisingly, stigma levels among nurses towards suicide who expressed dissatisfaction with their income were significantly lower than those of other nurses (as detailed in Table 3).

**TABLE 3:** Relationship of individual and social variables with the stigma of suicide in nurses.

Variable	Univariate model		Multiple model	
	B (95% Wald CI)	*p*	B (95% Wald CI)	*p*
Sex				
Male/Female	0.056 (0.013, 0.099)	0.011	0.038 (−0.004, 0.081)	0.077
Age	0.005 (0.002, 0.008)	0.002	0.011 (−0.002, 0.024)	0.101
Level of education				
Bachelor's degree/Masters	−0.009 (−0.086, 0.067)	0.810	−0.045 (−0.128, 0.038)	0.288
Satisfaction of income				
No/Yes	−0.052 (−0.12, 0.016)	0.132	−0.082 (−0.163, −0.002)	0.045
Type of employment				
Formal/New graduated	0.177 (0.075, 0.278)	0.001	0.141 (0.021, 0.261)	0.021
Informal/New graduated	0.186 (0.072, 0.299)	0.001	0.169 (0.045, 0.292)	0.007
Ward				
Emergency/Poisoning	0.049 (−0.025, 0.123)	0.194	0.02 (−0.055, 0.095)	0.600
ICU/Poisoning	0.055 (−0.026, 0.136)	0.186	0.021 (−0.06, 0.102)	0.604
Internal/Poisoning	0.014 (−0.058, 0.086)	0.702	−0.015 (−0.089, 0.059)	0.695
Suicide training				
No/Yes	0.027 (−0.016, 0.069)	0.219	0.04 (−0.006, 0.087)	0.085
Experience in the suicide ward				
No/Yes	0.034 (−0.004, 0.071)	0.076	0.016 (−0.023, 0.055)	0.420
Work experience	0.004 (0.001, 0.008)	0.007	−0.007 (−0.02, 0.007)	0.330
Number of shifts in a month	0.001 (−0.003, 0.005)	0.540	0.005 (0, 0.009)	0.060

Abbreviations: B, regression coefficient; CI, confidence interval.

### 3.6 Factors Associated With Suicide Knowledge in Physicians

The study employed generalised linear models to examine the associations between study variables and the level of suicide knowledge among physicians. Initially, a straightforward approach was used to fit the model, assessing each variable in isolation. Subsequently, all the variables were collectively included in a multiple model to explore their connections with suicide knowledge while accounting for the influence of other variables. Under the univariate model, no statistically significant relationships were observed between the factors and perceived stress at the 5% error level. However, when considering a 10% error level, it was revealed that physicians with contractual employment status exhibited significantly higher suicide knowledge compared to their peers.

In the multiple model, when controlling for other factors, physicians who expressed dissatisfaction with their income exhibited significantly greater suicide knowledge. Additionally, physicians with more extensive experience also exhibited higher levels of suicide knowledge compared to other physicians (as detailed in Table 4).

**TABLE 4:** Relationship of individual and social variables with knowledge of suicide in physician.

Variable	Univariate model		Multiple model	
	B (95% Wald CI)	*p*	B (95% Wald CI)	*p*
Sex				
Male/Female	0.002 (−0.138, 0.142)	0.975	−0.457 (−1.022, 0.107)	0.112
Age	0.001 (−0.005, 0.007)	0.720	−0.036 (−0.088, 0.017)	0.181
Satisfaction of income				
No/Yes	−0.072 (−0.223, 0.078)	0.348	0.584 (0.004, 1.164)	0.048
Type of employment				
Formal/New graduated	0.128 (−0.058, 0.314)	0.177	−0.07 (−0.812, 0.672)	0.853
Informal/New graduated	0.187 (−0.003, 0.377)	0.054	0.054 (−0.421, 0.528)	0.824
Suicide training				
No/Yes	−0.029 (−0.168, 0.11)	0.685	−0.6 (−1.364, 0.165)	0.124
Experience in the suicide ward				
No/Yes	0.113 (−0.04, 0.267)	0.148	−0.248 (−0.69, 0.194)	0.271
Work experience	0.005 (−0.003, 0.013)	0.240	0.057 (0.003, 0.111)	0.040
Number of shifts in a month	0.004 (−0.012, 0.021)	0.612	−0.014 (−0.058, 0.029)	0.513

Abbreviation: CI, confidence interval.

### 3.7 Factors Associated With Suicide Stigma in Physicians

The study employed generalised linear models to examine the connections between study variables and the level of suicide stigma among physicians. Initially, a simple approach was used to fit the model, analysing each variable individually. Subsequently, all the variables were collectively incorporated into a multiple model to explore their associations with suicide stigma while taking other variables into account. Based on the univariate model, it was evident that the stigma associated with suicide was notably higher among physicians who lacked training in dealing with suicidal patients. Additionally, physicians with no prior experience in working in suicide departments displayed higher levels of suicide stigma.

In the multiple model, suicide stigma was significantly lower in physicians who had no history of service in suicide departments when controlling for other factors. Interestingly, the average stigma score related to suicide was higher among physicians with prior experience in this field compared to their counterparts (as depicted in Table 5).

**TABLE 5:** Relationship of individual and social variables with the stigma of suicide in doctors.

Variable	Univariate model		Multiple models	
	B (95% Wald CI)	*p*	B (95% Wald CI)	*p*
Sex				
Male/Female	0.06 (−0.012,0.132)	0.100	−0.116 (−0.311,0.079)	0.244
Age	0.0001 (−0.004,0.003)	0.907	−0.018 (−0.036, −0.001)	0.043
Satisfaction of income				
No/Yes	0.064 (−0.012,0.14)	0.101	0.115 (−0.072,0.302)	0.229
Type of employment				
Formal/New graduated	−0.007 (−0.113,0.098)	0.891	0.103 (−0.145,0.351)	0.416
Informal/New graduated	0.092 (−0.016,0.2)	0.095	0.191 (−0.012,0.393)	0.065
Suicide training				
No/Yes	0.101 (0.031,0.17)	0.004	0.072 (−0.201,0.346)	0.605
Experience in the suicide ward				
No/Yes	0.099 (0.023,0.174)	0.011	−0.185 (−0.362, −0.008)	0.041
Work experience	0.0001 (−0.004,0.004)	0.935	0.021 (0.002,0.039)	0.027
Number of shifts in a month	0.004 (−0.004,0.013)	0.292	0.009 (−0.007,0.024)	0.285

Abbreviation: CI, confidence interval.

### 3.8 Correlation Between Knowledge and Stigma in Nurses and Physicians

**FIGURE 1:**
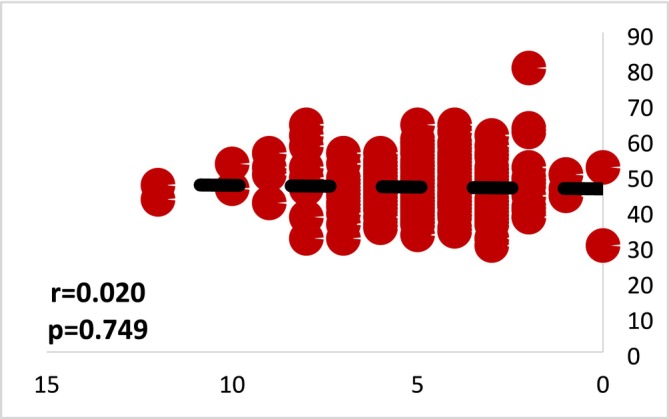
Correlation between knowledge and stigma of suicide in nurses (*r*: Spearman's rho correlation).

**FIGURE 2:**
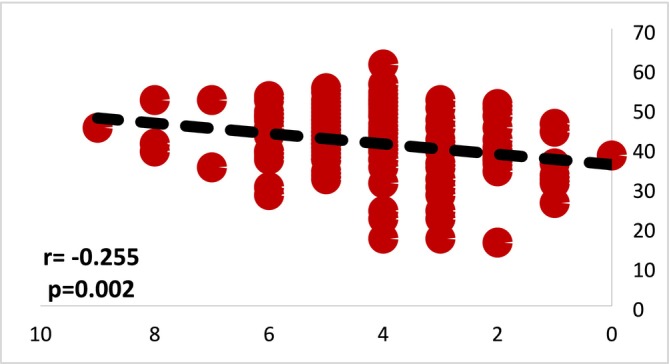
Correlation between knowledge and stigma of suicide in doctors (*r*: Spearman's rho correlation).

In this study, the association between suicide knowledge and suicide stigma in nurses was assessed using Spearman's correlation test. The results revealed no significant relationship between knowledge of suicide and the stigma associated with it (*p* = 0.749). When examining the correlation between suicide knowledge and suicide stigma in physicians, the study employed Spearman's correlation test as well. The findings indicated a significant increase in suicide stigma as suicide knowledge increased, with a negative correlation coefficient of −0.255. Figures 1 and 2 illustrate the correlation results between suicide knowledge and stigma among nurses and physicians.

## 4 Discussion

This study aimed to assess the level of stigma and suicide knowledge among physicians and nurses attending patients with a history of suicide attempts in medical educational hospitals in Babol City. The study found that the average suicide knowledge score for nurses was 4.65 ± 1.78, while the mean scores for general and specialist physicians were 4.51 ± 1.51 and 3.80 ± 1.58, respectively. Notably, there was a statistically significant difference between the suicide knowledge scores of physicians and nurses. While nurses scored higher than physicians in terms of suicide knowledge, the overall scores were still moderate, suggesting that there may be gaps in comprehensive understanding or application of this knowledge. Additionally, further training may be needed to address the emotional and attitudinal barriers, such as stigma, that healthcare workers, including nurses, face when dealing with suicidal patients (Betz et al. 2013; Fry et al. 2019; Jones et al. 2015).

The present study found that there was no significant relationship between knowledge of suicide and the stigma associated with it. These findings are similar to other research (Olibamoyo et al. 2020). Others found that there was no identified relationship between suicide literacy and suicide stigma among physicians (Maruf et al. 2022). The current study findings suggested that income satisfaction and the number of shifts per month are significant in nurses' knowledge of suicide. Yet other researchers pointed to marital status that was associated with suicide literacy (Arafat et al. 2022; Schernhammer and Colditz 2004). Our study showed that nurses' ages and education do not show any significant difference in suicidal knowledge, and these study findings are similar to a previous study (Arafat et al. 2022).

According to the findings, a large number of physicians and nurses had insufficient awareness of suicide and had unfavourable attitudes towards patients with a history of suicide attempts. Similar to the previous research, most physicians saw suicide as an existential crisis, but most nurses saw it as a moral transgression. In a different cultural context, Malaysian nurses' attitudes were in favour of accepting responsibility and receiving training in suicide prevention (Siau et al. 2019). Suicide prevention education should focus on improving the attitudes of non‐psychiatric nurses, as well as those with less experience or no experience in caring for suicidal patients since these groups tend to have more negative attitudes towards such patients. Additionally, training is recommended to enhance HCP's knowledge and skills in managing suicidal behaviour (Siau et al. 2019).

The research revealed that nurses exhibited higher levels of suicide stigma compared to both general and specialised physicians, with specialised physicians demonstrating the lowest levels of stigma among the groups. Nurses had statistically significantly higher suicide stigma ratings than physicians. These findings support earlier studies showing nurses' unfavourable views of suicidal people. It was previously understood that physicians had higher suicide literacy than nurses (Karakaya et al. 2023; Kumar et al. 2016), likely due to their more extensive and targeted training in mental health (Aldalaykeh et al. 2021), their responsibilities in assessing and managing suicidal patients, and their clinical experience dealing with high‐risk situations.

Some researchers believe clinicians' unfavourable views on suicide are linked to decreased empathy during patient interactions (Rukundo et al. 2022). Unfavourable attitudes towards suicide may be explained by societal factors such as religious disapproval (Radhakrishnan and Andrade 2012; Sudak et al. 2008), clinicians internalising the stigma of society towards suicide (Sudak et al. 2008) and considering suicide as a criminal violation (Ranjan et al. 2014).

The study also identified gender differences in attitudes towards suicidal patients, with male nurses exhibiting more negative attitudes than their female counterparts. Furthermore, nurses' employment status was associated with variations in attitudes towards individuals with a history of suicide attempts. Consistent with previous research, psychiatric nurses, those with more experience and those who had worked with suicidal patients were found to hold more favourable attitudes towards this population (Siau et al. 2019). Prior studies have also linked diminished empathy and professional burnout to increased cynicism and negative attitudes towards individuals with suicidal behaviour (Dyrbye et al. 2017; Yang et al. 2022). The observed discrepancies in findings may be attributed to cultural or religious differences across study samples. However, these results warrant further exploration of the psychological and psychosocial factors influencing HCP's attitudes towards suicide and suicidal patients.

Furthermore, the research revealed a significant relationship between suicide knowledge and a positive attitude among physicians dealing with patients with suicide attempts. As physicians' suicide knowledge increased, their attitudes became more positive. Contrastingly, another study showed no significant relationship between suicide knowledge and suicide stigma among Bangladeshi physicians (Maruf et al. 2022). The study also highlighted that physicians who had not received training for handling suicidal patients and those lacking experience in the field had higher levels of suicide stigma. This suggests that advanced degrees in the medical field alone do not significantly influence awareness and attitudes towards suicide, emphasising the need for specialised training focused on improved awareness and knowledge of suicide.

Although physicians had more professional experience and reported better awareness of suicidal behaviours, many held negative attitudes, viewing suicide as a “right,” a “justified solution,” or “unpreventable,” and believed that not all threats lead to attempts. These findings align with previous studies, which showed that physicians tend to have more negative attitudes towards suicide than nurses (Ghuloum et al. 2022; Nazli et al. 2022). Some researchers suggest physicians' lack of empathy during patient interactions could contribute to these negative attitudes (Rukundo et al. 2022). Socio‐cultural factors, such as the religious condemnation of suicide (Gearing and Alonzo 2018; Radhakrishnan and Andrade 2012), internalised social stigma (Sudak et al. 2008) and the view of suicide as a punishable offence (Ranjan et al. 2014) may also explain this perspective.

The research indicated that the variations in these findings could be attributed to factors such as the measurement tools used, cultural differences, sample size and measurement methods. In terms of demographic factors, the study found that older nurses had higher suicide knowledge, while nurses who were dissatisfied with their income had significantly lower suicide knowledge scores. However, the findings contradicted a study by Aldalaykeh et al., which showed no significant relationship between nurses' demographic characteristics and their attitudes towards suicidal patients (Aldalaykeh et al. 2021). This inconsistency might be due to cultural or religious diversity in the samples or the lack of suicide education and low levels of knowledge of the participants in their study.

### 4.1 Research Limitation

The study's cross‐sectional design means it captured data at a single point in time. While this allows for the identification of relationships between factors (e.g., experience, gender and suicide knowledge), it does not allow for conclusions about causality. Consequently, the researchers advise employing longitudinal or correlational approaches to find relationships in future studies. Additionally, the reliance on self‐reported data to assess suicide knowledge, attitudes and stigma introduces the possibility of response bias. HCPs may have underreported their stigma or overestimated their knowledge due to social desirability bias, particularly since attitudes towards suicide can be stigmatised. This could affect the accuracy of the findings, despite briefing the participants that their information was confidential. Lastly, the study focused on HCPs in a specific geographic location (northern Iran). This regional focus may limit the generalisability of the findings to other countries or regions with different cultural, religious or healthcare contexts. The sample may not fully represent the broader population of HCPs worldwide, potentially introducing regional or cultural bias in the attitudes and knowledge assessed. Longitudinal or experimental studies would be needed to determine how attitudes and knowledge change, particularly in response to educational interventions or shifts in clinical experience.

### 4.2 Implications for HCPs

This study highlights important gaps in suicide knowledge and the persistence of stigmatising attitudes among HCPs. While suicide literacy was associated with lower stigma among physicians, this relationship was not observed among nurses, suggesting that knowledge alone may not be sufficient to shift attitudes in nursing practice. These findings suggest the need for targeted, evidence‐based interventions to address stigma in clinical settings.

Research has shown that structured suicide prevention training programs can improve knowledge and reduce stigma among HCPs. For example, Applied Suicide Intervention Skills Training and Mental Health First Aid have been found effective in enhancing suicide literacy and fostering more compassionate responses to suicidal patients (Kavalidou et al. 2020; Cross et al. 2011). Integrating these programs into continuing education curricula for nurses may help bridge the gap between knowledge and practice.

Moreover, studies suggest that negative attitudes towards suicidal patients can be linked to emotional discomfort and a lack of preparedness in dealing with suicide crises (Chan et al. 2017; Sun et al. 2014). Implementing targeted training sessions that not only provide factual knowledge but also address emotional and attitudinal barriers—such as role‐playing exercises and exposure to lived experiences of suicide survivors—may help reduce stigma in nursing care.

The impact of stigma on patient care is well documented, with evidence indicating that stigmatising attitudes among HCPs can result in suboptimal care, reluctance to engage in suicide risk assessments and poorer therapeutic relationships (Pompili et al. 2013). To mitigate these risks, healthcare institutions should foster an organisational culture that promotes open discussions on suicide‐related attitudes and misconceptions. Reflective practice sessions, interdisciplinary team meetings and mental health consultation services may provide a supportive environment for professionals to challenge biases and improve their clinical approach to suicidal patients (Samuelsson and Asberg 2002).

Finally, institutional policies should prioritise mandatory suicide prevention training for all HCPs, particularly nurses, to ensure that suicide literacy translates into improved patient outcomes. Future research should evaluate the effectiveness of different training models in reducing suicide stigma among nurses and explore strategies to sustain long‐term attitudinal changes.

## 5 Conclusion

This study highlights important disparities in suicide knowledge and stigma among HCPs. While nurses demonstrated higher suicide knowledge than physicians, their levels of suicide stigma were significantly greater, suggesting that knowledge alone does not necessarily translate into reduced stigma. Among physicians, specialists had the lowest suicide knowledge scores but exhibited lower stigma compared to GPs and nurses. Additionally, more experienced physicians demonstrated higher suicide knowledge, whereas those without formal suicide management training reported greater stigma. The negative correlation between suicide knowledge and stigma among physicians suggests that enhancing suicide literacy may foster more compassionate attitudes. These findings suggest the need for integrating targeted, evidence‐based suicide prevention education into healthcare training programmes. Strengthening suicide literacy and addressing attitudinal barriers can ultimately improve patient care and contribute to more effective suicide prevention efforts within healthcare settings.

## Author Contributions

NStudy concept and design: H.O.T. and A.M. Acquisition, analysis or interpretation of data: Z.H.M., A.F., H.O.T. and A.M. Drafting of the manuscript: Z.H.M., N.K., M.A.K. and H.O.T. Critical revision of the manuscript for important intellectual content: M.Y., A.M., N.K., A.F., H.O.T., Z.H.M. and A.M. Statistical analysis: A.F.

## Ethics Statement

The conduct of this study was approved by the Ethics Committee of the Babol University of Medical Sciences, Babol, in Iran (IR.MUBABOL.REC.1401.198). All participants provided informed consent by clicking ahead from the participant information section of the survey to proceed with its completion. All methods employed in this study were conducted in accordance with the Declaration of Helsinki and its later amendments.

## Consent

The authors have nothing to report.

## Conflicts of Interest

The authors declare no conflicts of interest.

## Data Availability

The datasets used and/or analysed during the present study are available from the corresponding author upon reasonable request.

## References

[nop270182-bib-0001] Aldalaykeh, M. , M. M. Al‐Hammouri , J. Rababah , Y. Al‐Shannaq , and T. Al‐Dwaikat . 2021. “Knowledge of Jordanian Nurses and Attitudes Toward Patients With Suicidal Attempt.” Archives of Psychiatric Nursing 35, no. 6: 664–668. 34861962 10.1016/j.apnu.2021.10.004

[nop270182-bib-0002] Aldalaykeh, M. , H. Dalky , G. Shahrour , and M. Rababa . 2020. “Psychometric Properties of Two Arabic Suicide Scales: Stigma and Literacy.” Heliyon 6, no. 4: e03877. 32373752 10.1016/j.heliyon.2020.e03877 PMC7193320

[nop270182-bib-0003] Al‐Shannaq, Y. , and M. Aldalaykeh . 2021. “Suicide Literacy, Suicide Stigma, and Psychological Help Seeking Attitudes Among Arab Youth.” Current Psychology 42: 6532–6544. 34177209 10.1007/s12144-021-02007-9 PMC8214717

[nop270182-bib-0004] Al‐Shannaq, Y. , and M. Aldalaykeh . 2023. “Suicide Literacy, Suicide Stigma, and Psychological Help Seeking Attitudes Among Arab Youth.” Current Psychology 42, no. 8: 6532–6544. 10.1007/s12144-021-02007-9. 34177209 PMC8214717

[nop270182-bib-0005] Alshutwi, M. , M. Alawad , M. Alammari , et al. 2023. “Perceived Impact of Patients' Suicide and Serious Suicidal Attempts on Their Treating Psychiatrists and Trainees: A National Cross‐Sectional Study in Saudi Arabia.” BMC Psychiatry 23, no. 1: 607. 37596547 10.1186/s12888-023-05042-x PMC10439610

[nop270182-bib-0006] Arafat, S. Y. , F. Hussain , M. F. Hossain , M. A. Islam , and V. Menon . 2022. “Literacy and Stigma of Suicide in Bangladesh: Scales Validation and Status Assessment Among University Students.” Brain and Behavior: A Cognitive Neuroscience Perspective 12, no. 1: e2432. 10.1002/brb3.2432 PMC8785610 34856071

[nop270182-bib-0007] Asadiyun, M. , and S. Daliri . 2023. “Suicide Attempt and Suicide Death in Iran: A Systematic Review and Meta‐Analysis Study.” Iranian Journal of Psychiatry 18, no. 2: 191–212. 37383956 10.18502/ijps.v18i2.12370 PMC10293692

[nop270182-bib-0008] Askari, R. , H. F. Abarghouei , M. Heidarijamebozorgi , Z. Keyvanlo , and M. Kargar . 2021. “Job Burnout Among Nurses in Iran: A Systematic Review and Meta‐Analysis.” Nursing and Midwifery Studies 10, no. 2: 65–72.

[nop270182-bib-0009] Batterham, P. J. , A. L. Calear , and H. Christensen . 2013. “Correlates of Suicide Stigma and Suicide Literacy in the Community.” Suicide and Life‐Threatening Behavior 43, no. 4: 406–417. 23556504 10.1111/sltb.12026

[nop270182-bib-0010] Betz, M. E. , A. F. Sullivan , A. P. Manton , et al. 2013. “Knowledge, Attitudes, and Practices of Emergency Department Providers in the Care of Suicidal Patients.” Depression and Anxiety 30, no. 10: 1005–1012. 23426881 10.1002/da.22071 PMC4350671

[nop270182-bib-0011] Calear, A. L. , P. J. Batterham , and H. Christensen . 2014. “Predictors of Help‐Seeking for Suicidal Ideation in the Community: Risks and Opportunities for Public Suicide Prevention Campaigns.” Psychiatry Research 219, no. 3: 525–530. 25048756 10.1016/j.psychres.2014.06.027

[nop270182-bib-0013] Carpiniello, B. , and F. Pinna . 2017. “The Reciprocal Relationship Between Suicidality and Stigma.” Frontiers in Psychiatry 8: 35. 28337154 10.3389/fpsyt.2017.00035 PMC5340774

[nop270182-bib-0014] Chan, W. I. , P. J. Batterham , H. Christensen , and C. Galletly . 2017. “Suicide Literacy, Attitudes and Stigma in the Context of Schizophrenia: Evidence From a Community Survey.” Suicide and Life‐Threatening Behavior 47, no. 4: 435–447.

[nop270182-bib-0015] Cross, W. , M. M. Matthieu , J. Cerel , and K. L. Knox . 2011. “Proximate Outcomes of Gatekeeper Training for Suicide Prevention in the Workplace.” Suicide and Life‐Threatening Behavior 41, no. 1: 87–96. 18275372 10.1521/suli.2007.37.6.659

[nop270182-bib-0016] Dyrbye, L. N. , T. D. Shanafelt , C. A. Sinsky , et al. 2017. “Burnout Among Health Care Professionals: A Call to Explore and Address This Underrecognized Threat to Safe, High‐Quality Care.” NAM Perspectives 7, no. 7.

[nop270182-bib-0017] Fry, M. , K. Abrahamse , S. Kay , and R. M. Elliott . 2019. “Suicide in Older People, Attitudes and Knowledge of Emergency Nurses: A Multi‐Centre Study.” International Emergency Nursing 43: 113–118. 30711435 10.1016/j.ienj.2019.01.003

[nop270182-bib-0018] Gearing, R. E. , and D. Alonzo . 2018. “Religion and Suicide: New Findings.” Journal of Religion and Health 57, no. 6: 2478–2499. 29736876 10.1007/s10943-018-0629-8

[nop270182-bib-0019] Gholamrezaei, A. , R. Rezapour‐Nasrabad , M. Ghalenoei , and M. Nasiri . 2019. “Correlation Between Suicide Literacy and Stigmatizing Attitude of Nurses Toward Patients With Suicide Attempts.” Revista Latinoamericana de Hipertension 14, no. 3: 351–355.

[nop270182-bib-0020] Ghuloum, S. , Z. R. Mahfoud , H. Al‐Amin , T. Marji , and V. Kehyayan . 2022. “Healthcare Professionals' Attitudes Toward Patients With Mental Illness: A Cross‐Sectional Study in Qatar.” Frontiers in Psychiatry 13: 884947. 35651821 10.3389/fpsyt.2022.884947 PMC9148967

[nop270182-bib-0021] Jandial, R. , K. Subramanian , E. Subramaniam , and S. Balasundaram . 2021. “Literacy and Attitudes of Healthcare Professionals Regarding Suicide: A Review.” Annals of SBV 10, no. 2: 25.

[nop270182-bib-0022] Jones, S. , M. Krishna , R. G. Rajendra , and P. Keenan . 2015. “Nurses Attitudes and Beliefs to Attempted Suicide in Southern India.” Journal of Mental Health 24, no. 6: 423–429. 25993050 10.3109/09638237.2015.1019051

[nop270182-bib-0023] Jung, H. , K. von Sternberg , and K. Davis . 2017. “The Impact of Mental Health Literacy, Stigma, and Social Support on Attitudes Toward Mental Health Help‐Seeking.” International Journal of Mental Health Promotion 19, no. 5: 252–267.

[nop270182-bib-0024] Karakaya, D. , A. Özparlak , and M. Önder . 2023. “Suicide Literacy in Nurses: A Cross‐Sectional Study.” Journal of Clinical Nursing 32, no. 1–2: 115–125. 34985161 10.1111/jocn.16205

[nop270182-bib-0025] Kavalidou, K. , L. Smith , and K. Ioannidis . 2020. “Suicide Prevention Training in Healthcare Professionals: A Systematic Review.” International Journal of Environmental Research and Public Health 17, no. 2: 512.31947517

[nop270182-bib-0026] Kishi, Y. , H. Kurosawa , H. Morimura , K. Hatta , and S. Thurber . 2011. “Attitudes of Japanese Nursing Personnel Toward Patients Who Have Attempted Suicide.” General Hospital Psychiatry 33, no. 4: 393–397. 21762837 10.1016/j.genhosppsych.2011.02.005

[nop270182-bib-0027] Kumar, N. , R. Rajendra , S. M. Majgi , M. Krishna , P. Keenan , and S. Jones . 2016. “Attitudes of General Hospital Staff Toward Patients Who Self‐Harm in South India: A Cross‐Sectional Study.” Indian Journal of Psychological Medicine 38, no. 6: 547–552. 28031591 10.4103/0253-7176.194920 PMC5178039

[nop270182-bib-0028] Lathabhavan, R. , Z. H. Marznaki , M. M. Kaggwa , M. Darvishi , A. Haghighi , and M. Yıldırım . 2024. “Exploring the Relationship Between Peritraumatic Dissociative Experiences, Post‐Traumatic Stress Disorder, Stigma, and Fear: A Three‐Wave Study During the COVID‐19 Pandemic.” Archives of Psychiatric Nursing 51: 176–182. 39034076 10.1016/j.apnu.2024.06.025

[nop270182-bib-0029] Lew, B. , D. Lester , F. I. Mustapha , et al. 2022. “Decriminalizing Suicide Attempt in the 21st Century: An Examination of Suicide Rates in Countries That Penalize Suicide, a Critical Review.” BMC Psychiatry 22, no. 1: 424. 10.1186/s12888-022-04060-5. 35739483 PMC9219191

[nop270182-bib-0030] Manouchehri, A. , Z. H. Marznaki , L. M. Atim , M. Mohammadian amiri , and M. M. Kaggwa . 2022. “The Relationship Between Causes of Suicidal Attempts in Iran and Individual and Social Variables: A Retrospective Study.” BMC Psychiatry 22, no. 1: 780. 36503535 10.1186/s12888-022-04449-2 PMC9743690

[nop270182-bib-0031] Maruf, M. M. , F. R. Shormi , M. W. H. Sajib , et al. 2022. “Level and Associated Factors of Literacy and Stigma of Suicide Among Bangladeshi Physicians: A Cross‐Sectional Assessment.” Mental Illness 2022: 9914388. 10.1155/2022/9914388.

[nop270182-bib-0032] Masoomi, M. , S. Hosseinikolbadi , F. Saeed , V. Sharifi , A. H. Jalali Nadoushan , and S. Shoib . 2023. “Stigma as a Barrier to Suicide Prevention Efforts in Iran.” Frontiers in Public Health 10: 1026451. 36699938 10.3389/fpubh.2022.1026451 PMC9868841

[nop270182-bib-0033] Nazli, A. I. M. , Y. T. Ooi , D. Thyagarajan , and R. Jamaluddin . 2022. “Attitude Toward Suicidal Behavior: A Cross‐Sectional Study Among Health‐Care Professionals in Northwest Malaysia.” Malaysian Journal of Psychiatry 31, no. 1: 1–6.

[nop270182-bib-0034] Nebhinani, M. , N. Nebhinani , L. Tamphasana , and A. D. Gaikwad . 2013. “Nursing Students' Attitude Towards Suicide Attempters: A Study From Rural Part of Northern India.” Journal of Neurosciences in Rural Practice 4, no. 4: 400–407. 24347946 10.4103/0976-3147.120240 PMC3858758

[nop270182-bib-0035] Noorani, N. , K. Alavi , S. K. Malakooti , S. Salimi , and A. Jalali . 2017. “Mental Health Services Use Among People That Attempt Suicide by Taking a Drug Overdose During the Last Year Before Their Suicide Commission.” International Journal of Review in Life Sciences 7, no. 3: 80–85.

[nop270182-bib-0036] Olibamoyo, O. , O. Coker , A. Adewuya , O. Ogunlesi , and O. Sodipo . 2020. “Frequency of Suicide Attempts and Attitudes Toward Suicidal Behaviour Among Doctors and Nurses in Lagos, Nigeria.” South African Journal of Psychiatry 26: 1402. 10.4102/sajpsychiatry.v26i0.1402 PMC7433261 32832124

[nop270182-bib-0037] Ouzouni, C. , and K. Nakakis . 2009. “Attitudes Towards Attempted Suicide: The Development of a Measurement Tool.” Health Science Journal 3, no. 4: 222–231.

[nop270182-bib-0038] Özaslan, A. , and M. Yıldırım . 2021. “Internalized Stigma and Self Esteem of Mothers of Children Diagnosed With Attention Deficit Hyperactivity Disorder.” Children's Health Care 50, no. 3: 312–324.

[nop270182-bib-0039] Özaslan, A. , M. Yildirim , E. Guney , M. N. İlhan , and P. Vostanis . 2024. “Mental Health Problems and Help‐Seeking Behaviours of Syrian Refugee Adolescents: Mediating Role of Self‐Stigma.” Psychological Medicine 54, no. 4: 732–741. 37642171 10.1017/S0033291723002416

[nop270182-bib-0040] Pompili, M. , Z. Rihmer , M. Innamorati , et al. 2013. “Healthcare Professionals and Suicide Prevention: Training, Attitudes, and Knowledge.” Clinical Neuropsychiatry 10, no. 1: 20–26.

[nop270182-bib-0041] Radhakrishnan, R. , and C. Andrade . 2012. “Suicide: An Indian Perspective.” Indian Journal of Psychiatry 54, no. 4: 304–319. 23372232 10.4103/0019-5545.104793 PMC3554961

[nop270182-bib-0043] Ranjan, R. , S. Kumar , R. D. Pattanayak , A. Dhawan , and R. Sagar . 2014. “(De‐) Criminalization of Attempted Suicide in India: A Review.” Industrial Psychiatry Journal 23, no. 1: 4–9. 25535437 10.4103/0972-6748.144936 PMC4261212

[nop270182-bib-0044] Rasanah . 2020. “Iran's Increasing Suicide Rate.” https://rasanah‐iiis.org/english/monitoring‐and‐translation/reports/irans‐increasing‐suicide‐rate/.

[nop270182-bib-0045] Reavley, N. J. , and A. F. Jorm . 2014. “Associations Between Beliefs About the Causes of Mental Disorders and Stigmatising Attitudes: Results of a National Survey of the Australian Public.” Australian and New Zealand Journal of Psychiatry 48, no. 8: 764–771. 24658293 10.1177/0004867414528054

[nop270182-bib-0046] Rizzo, A. , M. Yıldırım , G. G. Öztekin , et al. 2023. “Nurse Burnout Before and During the COVID‐19 Pandemic: A Systematic Comparative Review.” Frontiers in Public Health 11: 1225431. 10.3389/fpubh.2023.1225431. 37732086 PMC10507882

[nop270182-bib-0047] Rukundo, G. Z. , E. K. Wakida , S. Maling , et al. 2022. “Knowledge, Attitudes, and Experiences in Suicide Assessment and Management: A Qualitative Study Among Primary Health Care Workers in Southwestern Uganda.” BMC Psychiatry 22, no. 1: 605. 10.1186/s12888-022-04244-z. 36096787 PMC9465925

[nop270182-bib-0048] Samuelsson, M. , and M. Asberg . 2002. “Training Programs for Suicide Prevention: Evidence From Swedish Healthcare Professionals.” European Psychiatry 17, no. 7: 400–406.

[nop270182-bib-0049] Schernhammer, E. S. , and G. A. Colditz . 2004. “Suicide Rates Among Physicians: A Quantitative and Gender Assessment (Meta‐Analysis).” American Journal of Psychiatry 161, no. 12: 2295–2302. 15569903 10.1176/appi.ajp.161.12.2295

[nop270182-bib-0050] Scocco, P. , E. Toffol , A. Preti , and Team, S.P . 2016. “Psychological Distress Increases Perceived Stigma Toward Attempted Suicide Among Those With a History of Past Attempted Suicide.” Journal of Nervous and Mental Disease 204, no. 3: 194–202. 26751731 10.1097/NMD.0000000000000457

[nop270182-bib-0051] Siau, C. S. , L.‐H. Wee , T. H. Adnan , S. H. Yeoh , K. Perialathan , and S. Wahab . 2019. “Malaysian Nurses' Attitudes Toward Suicide and Suicidal Patients: A Multisite Study.” Journal for Nurses in Professional Development 35, no. 2: 98–103. 30741918 10.1097/NND.0000000000000520

[nop270182-bib-0052] Sudak, H. , K. Maxim , and M. Carpenter . 2008. “Suicide and Stigma: A Review of the Literature and Personal Reflections.” Academic Psychiatry 32: 136–142. 18349334 10.1176/appi.ap.32.2.136

[nop270182-bib-0053] Sun, F. K. , A. Long , J. Boore , and L. I. Tsao . 2014. “The Attitudes of Nurses Towards Suicidal Behavior: A Literature Review.” Journal of Psychiatric and Mental Health Nursing 21, no. 5: 345–359.22340071

[nop270182-bib-0054] Taghva, A. , Z. Farsi , Y. Javanmard , A. Atashi , A. Hajebi , and M. Khademi . 2017. “Stigma Barriers of Mental Health in Iran: A Qualitative Study by Stakeholders of Mental Health.” Iranian Journal of Psychiatry 12, no. 3: 163–171. 29062367 PMC5640577

[nop270182-bib-0055] Tavakoli, S. , V. Sharifi , M. Taj , and M. R. Mohammadi . 2010. “Stigma of Depression and Its Relationship With Attitudes Toward Seeking Professional Help Among Students.” Advances in Cognitive Sciences 12, no. 3: 19–33.

[nop270182-bib-0056] Wasserman, D. , Z. Rihmer , D. Rujescu , et al. 2012. “The European Psychiatric Association (EPA) Guidance on Suicide Treatment and Prevention.” European Psychiatry 27, no. 2: 129–141. 10.1016/j.eurpsy.2011.06.003. 22137775

[nop270182-bib-0057] Yang, N. , Y. Zhang , Z. Liu , F. Wang , G. Yang , and X. Hu . 2022. “Influence of Social Workers' Empathy Ability on Suicidal Ideation of Cancer Patients.” Frontiers in Public Health 10: 925307. 35968492 10.3389/fpubh.2022.925307 PMC9364132

[nop270182-bib-0058] Ziapour, A. , F. Chirico , G. Nucera , et al. 2023. “Suicide Attempts, Suicide and Their Association With Socio‐Demographic Variables in Iran: A Retrospective, Registry‐Based, Cohort Study (2016–2021).” Disaster and Emergency Medicine Journal 8, no. 1: 27–32. 10.5603/DEMJ.a2023.0001.

